# Primary adenoid cystic carcinoma of the trachea: clinical outcome of 38 patients after interdisciplinary treatment in a single institution

**DOI:** 10.1186/s13014-019-1323-z

**Published:** 2019-07-04

**Authors:** Benjamin A. Högerle, Felix Lasitschka, Thomas Muley, Nina Bougatf, Klaus Herfarth, Sebastian Adeberg, Martin Eichhorn, Jürgen Debus, Hauke Winter, Stefan Rieken, Matthias Uhl

**Affiliations:** 1 Department of Thoracic Surgery, Thoraxklinik, Heidelberg University Hospital, Röntgenstraße 1, 69126 Heidelberg, Germany; 2 Institute of Pathology, Heidelberg University Hospital, Im Neuenheimer Feld 224, 69120 Heidelberg, Germany; 3 Translational Research Unit, Thoraxklinik, Heidelberg University Hospital, Member of the German Center for Lung Research, Röntgenstraße 1, 69126 Heidelberg, Germany; 4 Translational Lung Research Center (TLRC), Member of the German Center for Lung Research (DZL), Im Neuenheimer Feld 156, 69120 Heidelberg, Germany; 5 Department of Radiation Oncology, Heidelberg University Hospital, Im Neuenheimer Feld 400, 69120 Heidelberg, Germany; 6 National Center for Radiation Oncology (NCRO), Heidelberg Institute for Radiation Oncology (HIRO), 69120 Heidelberg, Germany; 7 Heavy Ion Therapy Center (HIT), Heidelberg University Hospital, 69120 Heidelberg, Germany; 8 Department of Radiation Oncology and Radiation Therapy, Heidelberg University Hospital, Im Neuenheimer Feld 400, 69120 Heidelberg, Germany

**Keywords:** Adenoid cystic carcinoma, Carcinoma of the trachea, Carbon ion, Radiation therapy, Multi-modal treatment, Tracheal tumor surgery

## Abstract

**Background:**

Primary adenoid cystic carcinomas (ACCs) of the trachea are rare tumors of the central bronchial system. In patients presenting with unresectable tumors, severe comorbidities, or incomplete surgical resection, definitive radiotherapy is currently the recommended treatment. Irradiation with carbon ions (C^12^) has shown promising local control (LC) and survival rates in cases of ACCs of the head and neck. No data on the therapeutic efficacy of C12 radiotherapy in treating tracheal ACC has been published.

**Methods:**

All patients with histologically confirmed ACC of the trachea treated with surgery and/or radiation treatment at Heidelberg University Hospital between 1991 and 2017 were included in this analysis. Patient and treatment characteristics, short- and long-term toxicity after radiotherapy, overall survival (OS), freedom from local progression (FFLP), and freedom from distant progression (FFDP) were prospectively acquired and retrospectively analyzed.

**Results:**

Thirty-eight patients (23 women and 15 men) with a median age of 51 were treated by surgery (*n* = 20) and/or radiotherapy with either C^12^ (*n* = 7) or photons (*n* = 24). Of these patients, 61% presented with locally advanced (stage 4) ACC. The median follow-up for all patients was 74.5 months. The 5-year OS for all patients was 95% (10-year: 81%). The 5-year FFLP and FFDP were 96% (10-year: 83%) and 69% (10-year: 53%), respectively. In patients who underwent surgery alone, the 5-year OS was 100% (10-year: 80%). The 5-year FFLP and FFDP were 100% (10-year: 100%) and 80% (10-year: 60%), respectively. In patients who underwent radiotherapy alone, the 5-year OS was 100% (10-year: 83%). The 5-year FFLP and FFDP were 88% (10-year: 44%) and 67% (10-year: 34%), respectively. In patients who received multi-modal treatment including surgery and adjuvant radiotherapy, the 5-year OS was 84% (10-year: 84%). The 5-year FFLP was 100% (10-year: 100%) and the 5-year FFDP was 65% (10-year, 65%).

**Conclusions:**

The long-term prognosis is favorable if surgery is performed. In cases of an incomplete resection, good OS can still be achieved following adjuvant radiotherapy. For radiotherapy, irradiation with C^12^ shows promising first results. However, more data is needed to prove the long-term advantage of C^12^ over photons.

**Trial registration:**

The ethics committee of the Heidelberg University Hospital approved the retrospective data analysis (S-174/2019).

## Background

Two thirds of all tracheal tumors are malignant. Of these, 75% are squamous cell carcinomas (SCCs) and 15% are adenoid cystic carcinomas (ACCs) [1, 2]. Typically, people in their fourth to sixth decade of life are affected. An overall 5-year survival rate of 52% and an overall 10-year survival rate of 29% are reported, which is better than the expected 5- and 10-year survival rates of patients with non-small cell lung cancer (NSCLC) [3].

ACCs are categorized as a subtype of NSCLC [4]. However, ACCs differ from NSCLC in many aspects. ACCs grow primarily in the tracheal lumen, have a slower growth rate, and rarely develop lymph node and distant metastases (M), even in advanced tumor stages. Based on these differences, Bhattacharyya proposed a modified TNM staging system [5] in which tumor size is the dominating prognostic factor in determining patient survival. This implies that an advanced tumor growth does not exclude a curative treatment approach.

The treatment of choice for ACC patients is the complete surgical resection [6]. In the case of incomplete resection (R1, R2), an adjuvant radiotherapy as a multi-modal treatment approach is recommended. Radiotherapy as a definitive therapeutic concept is suitable for patients presenting with an unresectable tumor and/or severe comorbidities. The application of photons is most common for radiotherapy. Recently, hadron irradiation with carbon ions (C^12^) has been established for clinical use with promising results, particularly for ACCs of the head and neck region and skull-based tumors [7–10]. Compared to photons, C12 offers a favorable dose distribution with the possibility of dose escalation and better biological effectiveness [11–13].

Due to the rareness of tracheal ACC, very little data from other centers have been published. Here, we present the data of 38 patients with histologically confirmed ACC of the trachea who were treated by surgery and/or radiotherapy with either C^12^ or photons at our center.

## Methods

All patients with histologically confirmed ACC of the trachea treated with surgery and/or radiation treatment at the Heidelberg University Hospital between 1991 and 2017 were included in this retrospective analysis. The characteristics of all patients are summarized in Table 1.

**Table 1:** Patient characteristics

Characteristic	Value (%)
Age, y	
Median	51
Range	19–80
Sex	
Female	23 (61)
Male	15 (39)
Tumor stage (Bhattachyryya 2004)	
Stage 1	5 (13)
Stage 2	5 (13)
Stage 3	5 (13)
Stage 4	23 (61)
UICC Characteristics of stage 4 tumors	23
T stage	
T1	0 (0)
T2	0 (0)
T3	2 (9)
T4	21 (91)
N stage	
N0	16 (70)
N1	4 (17)
No data	3 (13)
M stage	
M0	18 (78)
M1	5 (22)
N1 + M1	1 (4)
Treatment	
Surgery	7 (18)
Radiation therapy	18 (47)
C12	6 (33)
Photons	12 (67)
Surgery and radiation therapy	13 (34)
C12	1 (8)
Photons	12 (92)
No treatment	1 (3)
Resection status	
R0	9 (45)
R1	10 (50)
R2	1 (5)

*Abbreviations*: *C12* carbon ions

The overall survival (OS), freedom from local progression (FFLP), and freedom from distant progression (FFDP) were calculated according to the Kaplan-Meier method. Postoperative complications and short- and long-term toxicity after radiation therapy were evaluated using the 4.0 Common Terminology Criteria for Adverse Events (CTCAE) classification. The data was analyzed using IBM SPSS 25 Statistics (IBM, Armonk, NY, USA).

## Results

A total of 38 patients with histologically confirmed ACC of the trachea were included in this study. The data of 23 female and 15 male patients with a median age of 51 (range: 19–80) years at first diagnosis were evaluated. The TNM staging was provided by Bhattachyryya [5]. It was further modified by the authors of this article to include the factors of M and perineural invasion (Pn), which is characteristic of ACC and is associated with local tumor recurrence and impaired overall prognosis [14]. All TNM staging factors are defined in Table 2. The TNM staging system is summarized in Table 3.

**Table 2:** Definitions of TNM staging factors for primary ACCs of the trachea

Stages	Definitions
T-Stage	
T1	Primary tumor confined to trachea; size < 2 cm
T2	Primary tumor confined to trachea; size > 2 cm
T3	Spread outside the trachea but not to adjacent organs or structures
T4	Spread to adjacent organs or structures
Tx	Unkown or cannot be assessed
N-Stage	
N0	No evidence of regional nodal disease
N1	Clinical or histological evidence of regional nodal disease
Nx	Unknown or cannot be assessed
M-Stage	
M0	No evidence of distant metastases
M1	Clinical or histological evidence of distant metastases
Mx	Unknown or cannot be assessed
Pn-Stage	
Pn0	No histological evidence of perineural invasion
Pn1	Histological evidence of perineural invasion
Pnx	Unkown or cannot be assessed

**Table 3:** TNM staging system for primary ACCs of the trachea

	T-Stage	N-Stage	M-Stage	Pn-Stage
Stage 1	1	0	0	0
Stage 2	2	0	0	0
Stage 3	3	0	0	0
Stage 4	4	0	0	0
	any T	1	0	0
	any T	any N	1	0
	any T	any N	0	1

Of the patients, five were initially diagnosed with stage 1 ACC (13%), five with stage 2 ACC (13%), and five with stage 3 ACC (13%). Most of the patients (*n* = 23 / 61%) presented with locally advanced stage 4 ACC. In 21 of them, the tumor had spread to adjacent organs or structures (T4, 91%). However, lymph node metastases were clinically or histologically confirmed in only four patients (N1, 17%). No lymph node metastases were detected in 16 patients (N0, 70%; NX: *n* = 3 / 13%). Clinically or histologically confirmed M were present in five patients (M1, 22%; M0: *n* = 18 / 78%). Combined lymph node and M were diagnosed in one patient (4%). There was histological evidence of Pn in five patients (Pn1, 22%; Pn0: *n* = 20 / 87%; PnX: *n* = 4 / 17%).

### Treatment

Surgery was performed in 20 patients (53%). Eleven patients (55%) were diagnosed with stage 4 ACC (stage 1: n = 4 / 20%; stage 2: n = 4 / 20%; stage 3: *n* = 1 / 5%). All data is summarized in Table 4.

**Table 4:** Treatment characteristics

	Surgery alone	Multi-modal treatment		Radiotherapy alone	
		Surgery + C12	Surgery + photons	C12	Photons
Stage 1	2 (29)	0 (0)	2 (17)	0 (0)	0 (0)
Stage 2	1 (14)	0 (0)	3 (25)	1 (17)	1 (8)
Stage 3	0 (0)	0 (0)	1 (8)	0 (0)	4 (33)
Stage 4	4 (57)	1 (100)	6 (50)	5 (83)	7 (59)
Overall	7 (100)	1 (100)	12 (100)	6 (100)	12 (100)

The number of patients is presented for each stage according to the treatment method. The percentage is shown in brackets

A radical resection (R0) was achieved in nine patients (45%) (stage 1: n = 3 / 15%; stage 2: n = 1 / 5%; stage 4: *n* = 5 / 25%). An R1 situation remained in 10 patients (50%) (stage 1: n = 1 / 5%; stage 2: n = 3 / 15%; stage 3: n = 1 / 5%; stage 4: n = 5 / 25%). A macroscopic residue of the tumor (R2) had to be left in situ in one patient (5%; stage 2).

All patients with an incomplete surgical resection (R1, R2) underwent adjuvant radiotherapy as a multi-modal treatment approach (C^12^: *n* = 1, photons: *n* = 10). Furthermore, two R0-resected patients (10%) also received adjuvant radiotherapy because the tumor was close to the resection margin (C^12^: *n* = 0, photons: *n* = 2). Therefore, radiotherapy was performed in 13 (65%) of 20 patients following surgery (C^12^: n = 1, photons: *n* = 12).

In contrast, there was an interdisciplinary discussion about giving 18 patients (47%) definitive radiotherapy. In this group, 12 patients (67%) were diagnosed with stage 4 ACC (stage 1: n = 0 / 0%; stage 2: n = 2 / 11%; stage 3: *n* = 4 / 22%). Six patients underwent irradiation with C^12^ (33%), whereupon five were found to have stage 4 ACC (83%). One patient (17%) had undergone surgery for ACC in 1986 and was then diagnosed with recurrent stage 2 ACC 34 years following tumor resection. Twelve patients (67%) received irradiation with photons (stage 2: n = 1 / 8%; stage 3: n = 4 / 33%; stage 4: *n* = 7 / 59%). A palliative setting was recommended for one patient (no treatment, 3%).

In most cases, both variants of radiotherapy included a primary plan with 3D conformal radiotherapy, IMRT, or carbon ion beam therapy as well as a boost plan with 3D conformal radiotherapy, IMRT, brachytherapy, or carbon ion beam therapy delivered to the planning target volume (PTV) in fractions from 1.8 to 5 Gy (Gy). Overall, the median dose for the PTV1 (primary plan) was 50 Gy (range: 46–63). A median dose of 16 Gy (range: 0–74) was applied to the PTV2 (boost plan). Therefore, the overall median total dose was 66 Gy (range: 48–74.4). For the irradiation with C^12^ carbon ion boost, the dose to PTV1 (photons) was 50 Gy in median (range: 50–54) and the dose to the PTV2 (C^12^ carbon ion) was 24 Gy in median (range: 18–24). In the photon only group, the dose to PTV1 was also 50 Gy in median (range: 46–54), and the dose to the PTV2 was 16 Gy in median (range: 8–74). Two patients received treatment with 60Gy (RBE) and 63Gy (RBE) carbon ion beam only, respectively. Two other patients received, after the primary plan (PTV1) with 50Gy photons, a brachytherapy boost with an total dose of 15Gy in 3 fractions. The resulting total dose in the C^12^ group was 72 Gy in median (range: 60–74.4) and 66 Gy (range: 48–74) in the photon group. LEM 1 were used as the RBE model for the carbon ion beam therapy planning. Treatment calculation was performed with an α/β of 2Gy for organs at risk and the planning target volume. All data are summarized in Table 5.

**Table 5:** Radiation treatment

Carbon ion only (PTV1)					
*PTV1 (Gy RBE)*	*Dpf (Gy RBE)*	*PTV2 (Gy RBE)*	*Dpf (Gy RBE)*	*TD (Gy)*	*(n = 2)*
60	3	0	0	*60*	1
63	3	0	0	*63*	1
Photons (PTV1) with Carbon ion Boost (PTV2)					
*PTV1 (Gy)*	*Dpf (Gy)*	*PTV2 (Gy RBE)*	*Dpf (Gy RBE)*		*(n = 5)*
50	2	18	3	*68*	1
54	2	18	3	*72*	1
50	2	24	3	*74*	2
50.4	1.8	24	3	*74.4*	1
Photons (PTV1) with Brachytherapy Boost (PTV2)					
*PTV1 (Gy)*	*Dpf (Gy)*	*PTV2 (Gy)*	*Dpf (Gy)*		*(n = 2)*
50	2	15	5	*65*	2
Photons only (PTV1 and PTV2)					
*PTV1 (Gy)*	*Dpf (Gy)*	*PTV2 (Gy)*	*Dpf (Gy)*		*(n = 22)*
46	2	10	2	*56*	1
46	2	14.4	1.8	*60.4*	1
48	2	–	–	*48*	1
50	2	–	–	*50*	1
50	2	8	2	*58*	2
50	2	13.8	2.3	*63.8*	1
50	2	16	2	*66*	8
50	2	22	2	*72*	2
50	2	24	2	*74*	1
50.4	1.8	9	1.8	*59.4*	3
54	1.8	72 (SIB)	2,4 (SIB)	*72*	1

*Abbreviations*: *PTV1* planning target volume 1 = primary plan, *PTV2* planning target volume 2 = boost plan, *Gy* Gray, *RBE* relative biological effectiveness, *Dpf* Dose per fraction, *TD* total dose, *SIB* simultaneous integrated boost

### Survival and local control

The median follow-up time for all patients was 74.5 months. The median follow-up time for patients who underwent surgery alone was 185 months. The median follow-up time for patients who underwent surgery and adjuvant radiotherapy was 123 months. In contrast, the median follow-up times for patients after definitive C^12^ treatment and photon treatment were 15.5 and 60 months, respectively. The 5-year OS of all patients was 95% (10-year: 81%). The 5-year FFLP was 96% (10-year: 83%), and the 5-year FFDP was 69% (10-year: 53%).

In patients who underwent surgery alone, the 5-year OS was 100% (10-year: 80%). The 5-year FFLP was 100% (10-year: 100%) and the 5-year FFDP was 80% (10-year: 60%). In patients who underwent radiotherapy alone, the 5-year OS was 100% (10-year: 83%). The 5-year FFLP and FFDP were 88% (10-year: 44%) and 67% (10-year: 34%), respectively. The longest follow-up period for C^12^ irradiation alone was 20 months. After this period, no patient had developed local or distant progression (FFLP/FFDP: 100%). Compared to photon irradiation alone, FFLP was also 100% after 21 months (FFDP: 91%). The longest follow-up period for photon irradiation alone was 11.3 years. After this period, FFLP was 43%. FFDP was 38% after 11.3 years.

In patients who received multi-modal treatment including surgery and adjuvant radiotherapy, the 5-year OS was 84% (10-year: 84%). The 5-year FFLP was 100% (10-year: 100%), and the 5-year FFDP was 65% (10-year: 65%). One patient underwent surgery and adjuvant C^12^ treatment. The follow-up period was 20 months. After this period, the patient had not developed any FFLP (FFDP). Compared to surgery and photon irradiation, FFLP was also 100% after 20 months (FFDP: 91%). The longest follow-up period for multi-modal treatment with photon irradiation was 12.3 years. After this period, FFLP was 75%. FFDP was 61% after 12.3 years.

**Fig. 1:**
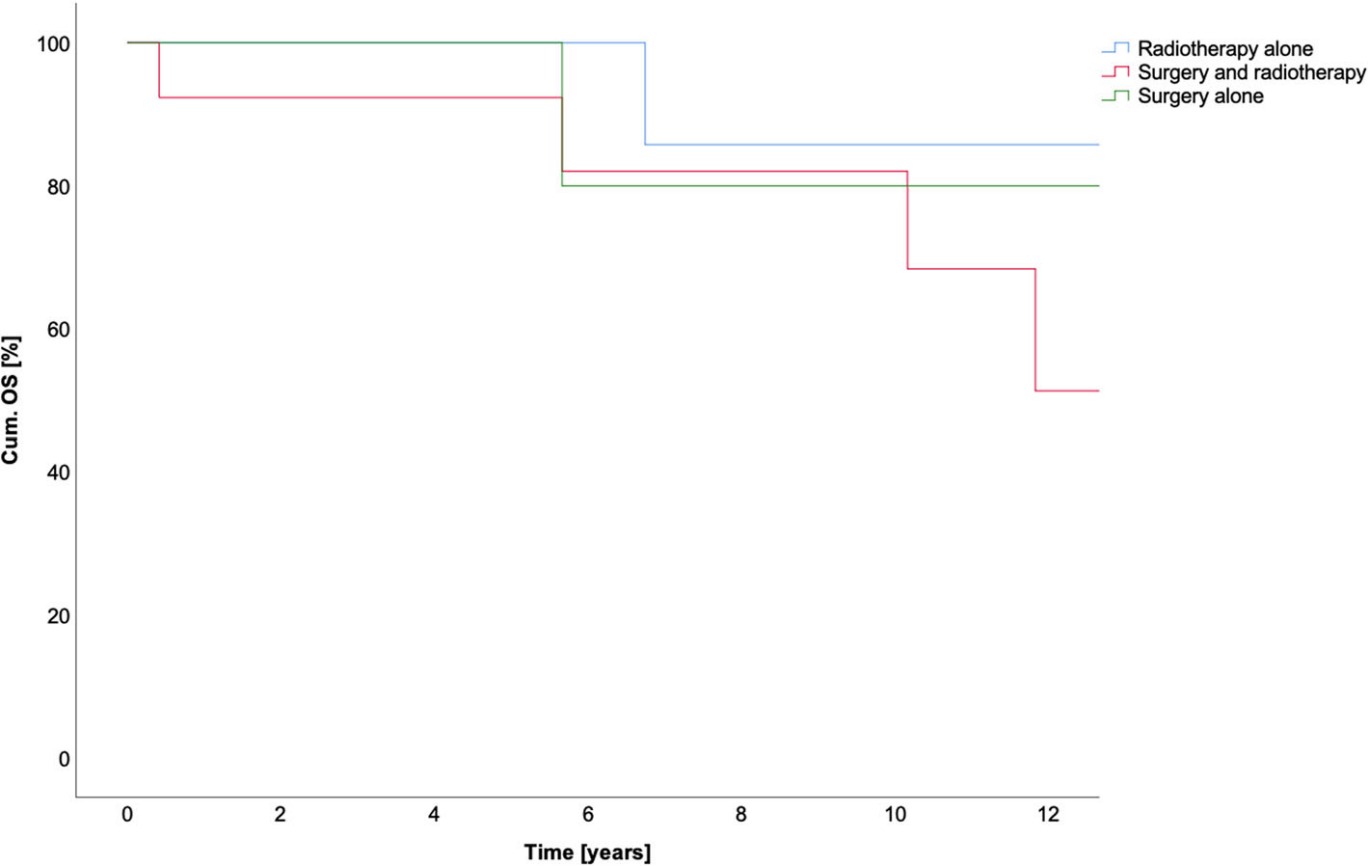
Cumulative overall survival (OS) in years

**Fig. 2:**
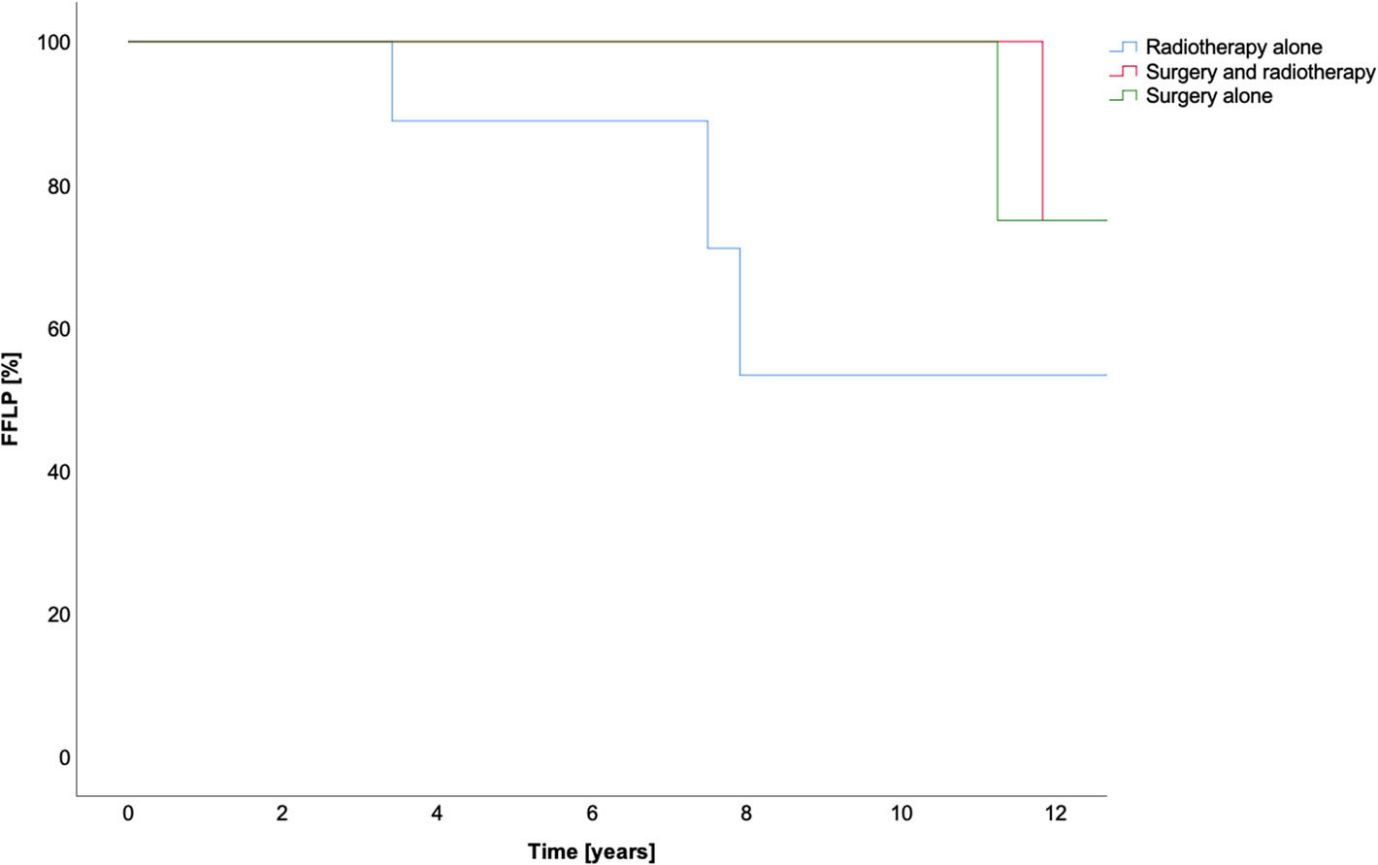
Freedom from local progression (FFLP) in years

**Fig. 3:**
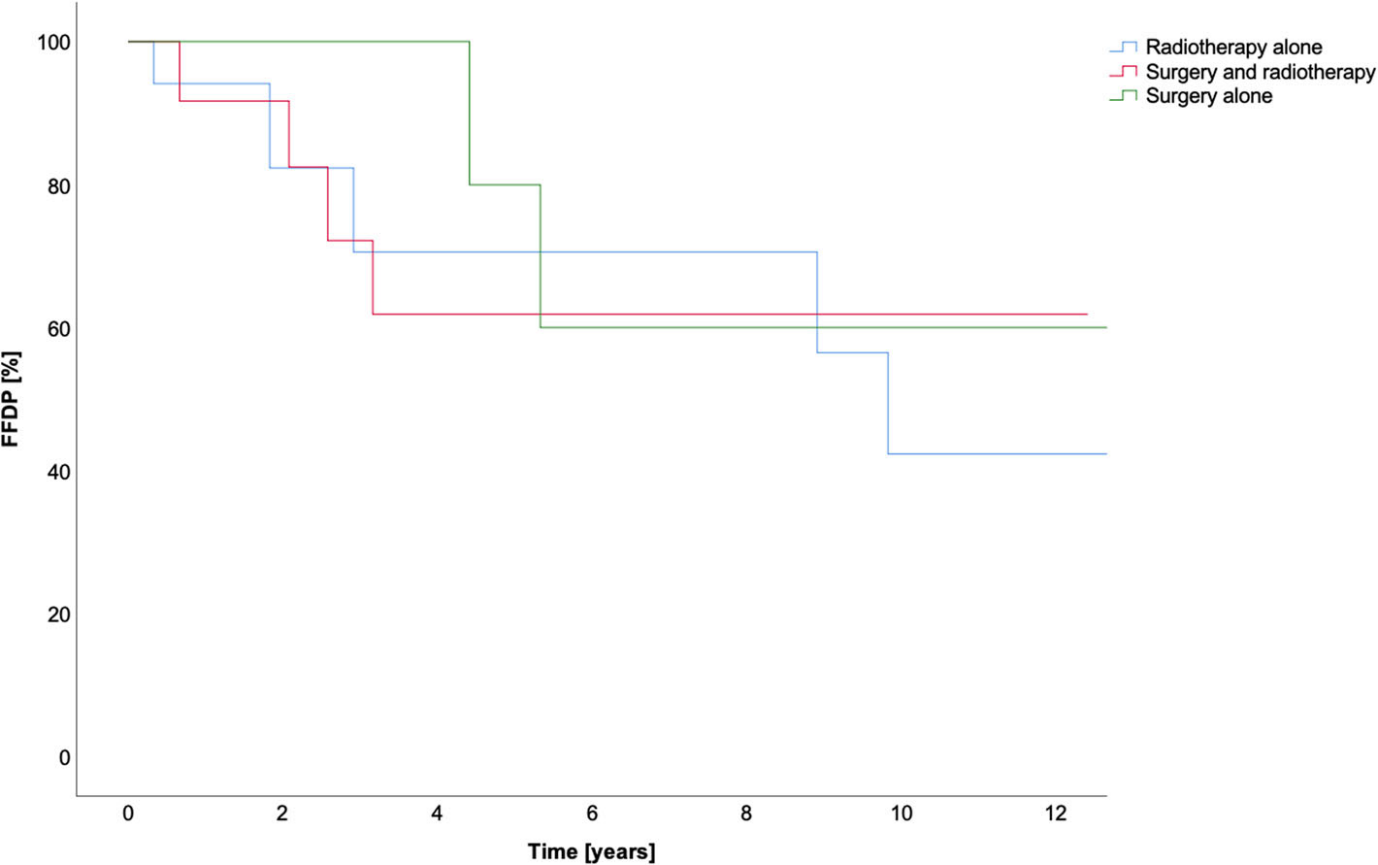
Freedom from distant progression (FFDP) in years

All data is summarized in Table 6. All results for OS in the different treatment groups are summarized in Fig. 1. The FFLP and the FFDP are shown in Figs. 2 and 3, respectively.

**Table 6:** Treatment method and survival

		Surgery alone	Multi-modal treatment		Radiotherapy alone		Overall
			Surgery + C12	Surgery + photons	C12	Photons	
OS	1-year	100	100	92	100	100	95
	5-year	100	n.a.	92	n.a.	100	95
	10-year	80	n.a.	82	n.a.	83	81
FFLP	1-year	100	100	100	100	100	100
	5-year	100	n.a.	100	n.a.	86	96
	10-year	100	n.a.	100	n.a.	43	83
FFDP	1-year	100	100	91	100	91	94
	5-year	80	n.a.	61	n.a.	76	69
	10-year	60	n.a.	61	n.a.	38	53

*Abbreviations*: *FFDP* freedom from distant progression, *FFLP* freedom from local progression, *n.a*. not available, *OS* overall survival

### Toxicity

After radiotherapy, acute toxicity was limited to CTCAE grade 1 in 16 patients (52%). One patient (3%) showed grade 2 toxicity, and one patient (3%) showed grade 3. No toxicity data were available for 13 patients (42%). Dysphagia, erythema, and mucositis were the most commonly observed symptoms and were well treatable. After C12 radiotherapy (*n* = 7), mainly CTCAE grade 1 toxicity was detected (5 patients / 72%). In one case (14%), the food intake was aggravated by severe stomatitis (CTCAE grade 4). There were no data available for one patient (14%). After irradiation with photons, 11 cases of CTCAE grade 1 toxicity (46%) and one case of grade 2 toxicity (4%) were documented (no data available for 12 patients / 50%). One female patient (4%) was diagnosed with breast cancer 16 years after photon irradiation.

## Discussion

### Survival and local control

ACCs arise most often in the salivary glands. In cases of the advanced tumor stage (T4), incomplete resection (R1, R2), or perineural invasion (Pn1), studies suggest adjuvant radiotherapy after surgery. In patients presenting with an unresectable tumor and/or severe comorbidities, definitive radiation therapy is indicated [15]. After multi-modal treatment, the 5- and 10-year OS was 94 and 91%, respectively. After definitive radiotherapy, the 5- and 10-year OS was up to 56 and 43%, respectively [16, 17].

ACC of the trachea is a very rare and slow-growing cancer that arises from the mixed seromucinous glands in the trachea. Therefore, only a few retrospective studies (particularly after radiation treatment) have been published to date. The published studies showed a good 5-year OS rate (> 70%) [18, 19]. These results are comparable with the OS in our study (5-year OS: 95%; 10-year OS: 81%). A complete R0 resection is considered the gold standard in treating tracheal ACC. Due to the infiltrative growth of ACCs into the surrounding tissue, incomplete resection margins are often observed following surgery. According to the literature, positive resection margins after surgery occur in 8–82% of all cases [6, 19, 20]. In our study, 55% (*n* = 11) of the operated patients had a microscopic (R1) or macroscopic residuum (R2). Although two published studies showed no significant difference in the OS of patients following completely or incompletely resected ACCs, these results must be discussed critically [1, 18]. Both published studies are based on a retrospective analysis of a few patients with ACC. In addition, the rate of adjuvant radiotherapy was different in these two studies. Adjuvant radiotherapy was performed in 75% / 100% of patients after incomplete resection. Only 33% / 0% of the patients received postoperative radiotherapy in the group of completely resected patients [1, 18]. In our study, 100% of the patients with incomplete resection (R1/2) received adjuvant radiotherapy and 22% with complete resection (R0) received adjuvant radiotherapy. Therefore, postoperative radiation after incomplete resection may be a possible explanation for the non-significant differences in OS following incomplete resection versus complete resection. In contrast, published data from the Massachusetts General Hospital in Boston showed survival benefit in patients with negative resection margins. In total, 91% of the patients had positive margins (tracheal, radial, or both), and only 9% had negative margins [19]. Furthermore, radiation treatment was confirmed in only 82% of the patients, while 17 patients with positive margins received no adjuvant radiation treatment. In our study, we found no significant prognostic factors for survival and/or LC. The small number of patients, the inhomogeneous treatment, and the different follow-up times of the subgroups could explain this. Other authors have identified tumor size, tumor location, age, surgery as an initial treatment, Pn, type of radiation, and radiation dose as possible prognostic factors for survival and/or LC [19–21]. Most of the patients in the current study presented with locally advanced, stage 4 ACC (61%). Case reports suggest argon plasma coagulation (APC) and chemotherapy as alternative treatment options [22, 23]. However, APC is only suitable for the palliative tumor stage, and chemotherapy alone seems to be ineffective [24]. Previous studies recommend surgery as the best treatment of choice [1, 6].

However, in functionally and/or technically inoperable patients, definitive radiotherapy is a good therapeutic alternative. Some studies have shown significantly worse outcomes with only 12–17% 5-year survival rates in patients after definitive radiation treatment compared to surgery [25]. However, French data from 2018 show no significant difference between operated and non-operated patients, with 5-year survival rates of 82 and 86% [21]. In the current study, the 5- and 10-year OS rates were not significantly different between surgery followed by adjuvant radiation treatment (5-year: 92%, 10-year: 82%) and definitive radiotherapy (5-year: 100%, 10-year: 83%). Furthermore, the 5-year-FFLP was not significantly improved in patients who underwent definitive surgery (100%) compared to those who underwent radiotherapy alone (87%). But again, the results have to be considered with caution. Due to the retrospective evaluation of only a few patients and the different follow-up times of the sub-groups, there may be a bias in favor of radiotherapy. Furthermore, as patients with advanced tumor stages and a poor Karnofsky index are treated with definitive radiation, there is also a possible survival bias in favor of surgery.

Particularly in patients who present with inoperable tracheal ACC, dose escalation with C^12^ is an option to improve tumor control, OS, and side effects. In addition to chordomas and chondrosarcomas, ACCs are one of the tumor entities that may benefit from escalation therapy with protons, neutrons, or C^12^. Furthermore, prospective studies have shown an advantage of hadrons over photons for the treatment of ACC originating in the salivary glands [26]. Data from 58 patients with ACC of the head and neck showed significantly better LC, progression-free survival (PFS), and OS at 5 years in the group with C^12^ boosts (59.6, 48.4, and 76.5%, respectively) compared to the photon only group (39.9, 27, and 58.7%, respectively) [27]. The median follow-up was 74 months in the C^12^ group and 63 months in the photon group. Overall, 90% of patients in the C12 group and 94% of those in the photon group had a T4 tumor. All patients had macroscopic residual tumors at the start of treatment. There was no significant difference in OS between patients who had subtotal resection and inoperable ACC [27]. Our results confirm the advantage of C^12^ irradiation compared to photon irradiation with regard to improved local (FFLP) and distant (FFDP) control. The physical and biological advantages of carbon ion beam therapy allow a dose escalation in the tumor (cumulative median dose in the current study: carbon ions: 72 Gy, photons: 66 Gy) without higher doses to the surrounding normal tissue. Therefore, the tumor control probability (TCP) is higher without increasing the normal tissue complication probability (NTCP). In addition, the higher relative biological effectiveness (RBE) yields to heavier DNA damage [28].

### Toxicity

Overall, radiotherapy was well tolerated. The acute toxicities were mainly limited to CTCAE grades 1 and 2 with favorable outcomes. No ulceration, fistulation, or necrosis was reported after radiation treatment. Sixteen years after photon irradiation, one patient (3%) was diagnosed with a secondary malignant tumor. This might be a long-term complication after radiotherapy, as the risk for secondary malignant tumors is known to be increased [29]. However, breast cancer is the most common tumor in women. The lifetime risk for breast cancer is much higher compared to the risk for secondary malignancy after radiation treatment. Therefore, a random occurrence of breast cancer cannot be excluded. It can be speculated that the incidence of secondary malignancies might be decreased by using C^12^ due to the physical advantages with lower integral doses in the normal tissue compared to radiation treatment with photons.

## Limitations

ACCs are very rare tumors of the central bronchial system. The present study retrospectively evaluates the relatively small number of 38 patients presenting with a primary ACC of the trachea. Due to the heterogeneity of the group and the different follow-up times of the sub-groups, the Kaplan–Meier method is susceptible to bias. Furthermore, comparison between the groups is difficult (for example, definitive surgery versus definitive radiotherapy or irradiation with C^12^ versus irradiation with photons). As ACCs are characterized by a slow growth rate, short follow-up times, particularly for the C^12^ group, may lead to an underestimated progression rate of the tumor. However, a longer follow-up and a bigger sample size are needed to minimize potential errors.

## Conclusions

Primary ACC of the trachea differs from NSCLC in many ways. Although most patients are first diagnosed as being in an advanced tumor stage, the long-term prognosis is favorable if surgery is performed. In cases of an incomplete resection, good OS can still be expected if an adjuvant radiotherapy as part of a multi-modal treatment approach was employed. In patients presenting with an unresectable tumor and/or severe comorbidities, definitive radiotherapy is indicated. For radiotherapy, irradiation with C^12^ shows promising first results with regard to OS and local (FFLP) and distant (FFDP) control compared to standard irradiation with photons. However, more data is needed to prove the long-term advantages of C^12^ over photons.

## Data Availability

The datasets used and/or analyzed during the current study are available from the corresponding author on reasonable request.

## Abbreviations

ACC: Adenoid cystic carcinoma
APC: Argon plasma coagulation
CTCAE: Common Terminology Criteria for Adverse Events
FFDP: Freedom from distant progression
FFLP: Freedom from local progression
IMRT: Intensity-modulated radiation therapy
LC: Local control
M: Distant metastases
NSCLC: Non-small cell lung cancer
NTCP: Normal tissue complication probability
OS: Overall survival
PFS: Progression-free survival
Pn: Perineural invasion
PTV: Planning target volume
RBE: Relative biological effectiveness
SCC: Squamous cell carcinomas
TCP: Tumor control probability
